# Subtle Effects of Biological Invasions: Cellular and Physiological Responses of Fish Eating the Exotic Pest *Caulerpa racemosa*

**DOI:** 10.1371/journal.pone.0038763

**Published:** 2012-06-11

**Authors:** Serena Felline, Roberto Caricato, Adele Cutignano, Stefania Gorbi, Maria Giulia Lionetto, Ernesto Mollo, Francesco Regoli, Antonio Terlizzi

**Affiliations:** 1 Dipartimento di Scienze e Tecnologie Biologiche ed Ambientali, Università del Salento, CoNISMa, Lecce, Italy; 2 Istituto di Chimica Biomolecolare, CNR, Pozzuoli, Naples, Italy; 3 Dipartimento di Scienze della Vita e dell’Ambiente, Università Politecnica delle Marche, Ancona, Italy; University of Connecticut, United States of America

## Abstract

The green alga *Caulerpa racemosa* var. *cylindracea* has invaded Mediterranean seabed including marine reserves, modifying the structure of habitats and altering the distributional patterns of associated organisms. However, the understanding of how such invasion can potentially affect functional properties of Mediterranean subtidal systems is yet to be determined. In this study, we show that *C. racemosa* changes foraging habit of the native white seabream, *Diplodus sargus*. In invaded areas, we found a high frequency of occurrence of *C. racemosa* in the stomach contents of this omnivorous fish (72.7 and 85.7%), while the alga was not detected in fish from a control area. We also found a significant accumulation of caulerpin, one of the main secondary metabolites of *C. racemosa*, in fish tissues. The level of caulerpin in fish tissues was used here as an indicator of the trophic exposure to the invasive pest and related with observed cellular and physiological alterations. Such effects included activation of some enzymatic pathways (catalase, glutathione peroxidases, glutathione S-transferases, total glutathione and the total oxyradical scavenging capacity, 7-ethoxy resorufin *O*-deethylase), the inhibition of others (acetylcholinesterase and acylCoA oxidase), an increase of hepatosomatic index and decrease of gonadosomatic index. The observed alterations might lead to a detrimental health status and altered behaviours, potentially preventing the reproductive success of fish populations. Results of this study revealed that the entering of alien species in subtidal systems can alter trophic webs and can represent an important, indirect mechanism which might contribute to influence fluctuations of fish stocks and, also, the effectiveness of protection regimes.

## Introduction

One of the main limits of spatially explicit forms of marine conservation (e.g, Marine Protected Areas) is that they fail to offer any protection from several major threats, acting often in a synergistic way outside their boundaries. Such threats include coastal modifications and subsequent changes in local hydrodynamic and sedimentary regimes, chemical pollution, disease epidemics and the spreading of exotic species [1], [2].

Alien species can cause severe changes in ecosystem’s functioning and are currently recognized as principal agents of global change [3]–[5]. About 955 alien species are reported in the Mediterranean basin [6]. Among these, the green algae *Caulerpa racemosa* has attracted great attention because of the significant sea-bottom landscape change induced in the last decades in the Mediterranean. Native from the south-western coast of Australia *C. racemosa* is present in the most part of Mediterranean Sea where has invaded also many Marine Protected Areas (MPAs) [e.g. 7].

The impact caused by the *C. racemosa* invasion is mainly due to increased siltation of bottom deriving from the high percentage of fine sediment retained from its stolons [8]. The extensive and uniform mats formed by *Caulerpa* spp., however, have also direct effects on feeding habit of demersal species [9], [10] since sandy or rocky substrates are less accessible with consequent alteration of predator-prey interactions [11] and potential decline of fish populations [12].

*Caulerpa racemosa* main secondary metabolites are considered among the factors contributing to its invasion potential [13]. Several studies have attempted to unravel the exact biological role of these compounds. Although the toxicity of caulerpenyne has been established [14]–[17], caulerpin and caulerpicin have been described in some studies as toxic [18], [19], but evidence from other studies indicates that they have only low or no acute toxicity [20], [21].

A preliminary investigation was carried out along the Apulian coasts (Northern Ionian Sea, SE Italy) during the summer of 2008 with the aim to evaluate the occurrence and the extent of the interaction between the invasive seaweed and the endemic white sea bream, *Diplodus sargus* [22]. This study showed, for the first time, that *D. sargus* has introduced *C. racemosa* in its diet accumulating one of its secondary metabolites, namely the alkaloid caulerpin, in several tissues [22].

Significant correlations among caulerpin tissue load and fish condition factor and hepatosomatic index were obtained, suggesting a possible detrimental effect of the dietary exposure to *C. racemosa* on *D. sargus* [22].

Also, glutathione peroxidase and catalase activity were significantly correlated with caulerpin concentration, indicating, as suggested by other studies [10], [17] the ability of *Caulerpa* spp. to increase production of ROS (Reactive Oxygen Species), leading to possible oxidative damage in fish [22].

Relationships between subcellular mechanisms of algal metabolites and indirect effects on marine biodiversity have seldom been investigated. In light of results obtained in [22], this study aimed at investigating the effects of such a new trophic interaction, by measuring toxicological responses at several biochemical and physiological levels in organism living in invaded and non-invaded environments. By conjugating organic chemistry, ecotoxicology and ecology, this study attempts to elucidate potential impact of *C. racemosa* on *D. sargus,* providing new insights into cellular mechanisms by which biological invasions can affect marine biodiversity and, hence, the effectiveness of protection regimes.

## Materials and Methods

### Study Area and Sampling

**Figure 1:**
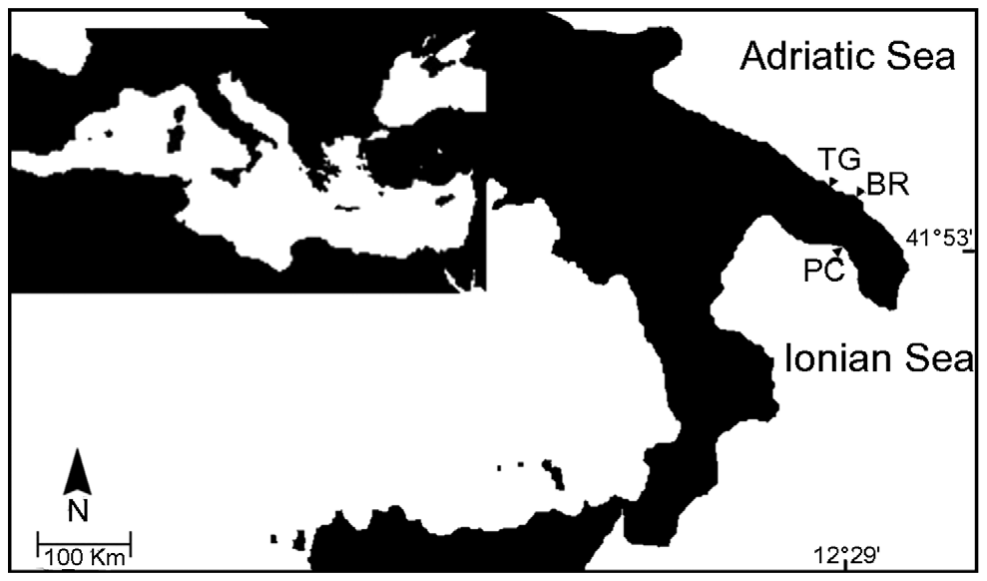
Sampling locations. Porto Cesareo (PC), Brindisi (BR) and Torre Guaceto (TG).

Fish were sampled over the period of one month (from late September to the end of October, with a water temperature of 24°C) in 3 locations along the Apulia coast varying in terms of presence and abundance of *C. racemosa*. These locations include the coast between Brindisi and Lecce (BR), and the Marine Protected Areas (MPAs) of Porto Cesareo (PC) and Torre Guaceto (TG) (Figure 1). The two MPAs are not interested by relevant sources of chemical pollution as supported by long-term biomonitoring projects [23], [24].

The 3 locations are characterized by the same rocky substrate, dropping from the water surface to about 8–16 m depth on sand. The compositional structure of the rocky sessile assemblages is comparable among locations and composed mainly by encrusting calcified red algae (including *Lithophyllum* sp. *Mesophyllum alternans* and *Peyssonnelia* spp.), filamentous dark algae (mostly red algae belonging to the order Ceramiales), articulated corallines (*Amphiroa rigida*, *Corallina elongata*), erect algae (*Halimeda tuna*, *Hypnea* sp., *Wrangelia verticillata*, *Laurencia* complex and the order Dyctiotales), filamentous green algae (*Bryopsis* sp., and the order Cladophorales), sponges (*Aplysina aerophoba*, *Chondrilla nucula*, *Chondrosia reniformis, Cliona* spp., *Crambe crambe*, *Ircinia spp.*, *Phorbas* spp., *Spirastrella cunctatrix*) and encrusting bryozoans (*Reptadeonella violacea* and *Schizobrachiella sanguinea* ) [25], [26].

The only differences among the three areas are due to the relative abundance of some species, that is algal turf and branched-erect algae showing a significant higher cover at the well-unforced protected area (TG) than at weakly-unforced (PC) and unprotected (BR) locations [27].

The presence of *C. racemosa* in PC is only occasional while BR and TG are invaded locations, characterised by the massive presence of the alga.

For each location, 15 individuals of *D. sargus* with mean weight of 373 g (SE=±27) and mean total length of 25 cm (SE=±0.6) were sampled early in the morning, by spearing. Once speared, individuals were immediately stored in a polystyrene box containing dry ice until transportation to the laboratory, where they were measured and weighed. Liver, spleen, gills and muscle were then excised, frozen in liquid nitrogen and maintained at −80°C till processed for analyses. Stomachs were removed by severing the oesophagus near the buccal cavity and the intestine just anterior to the pyloric caeca and their contents were preserved in 70% ethyl alcohol. Red muscle was also dissected and stored at −20°C until chemical analysis.

For each individual the condition factor (CF), hepatosomatic index (HSI) and gonadosomatic index (GSI) were calculated as follows:
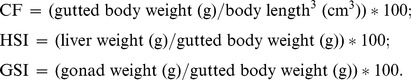



All experiments were carried out in accordance with the European Committee Council Directive (86/609/EEC) and with the Italian animal welfare legislation (D.L. 116/92). The experimental fishing activity performed within Marine Protected Areas were in strict accordance with the authorizations provided by the directors of MPAs of Torre Guaceto (Protocol n. 1947/PM/09, Sept, 14, 2009) and Porto Cesareo (Prot. R015, Oct, 15, 2009) on behalf of the Italian Ministry for the Environment, Land and Sea.

### Gut Content Analysis

Stomach contents were sorted under magnification and identified to the lowest possible taxonomic level, depending on the type and digestion level of each prey item. The wet weight of each item was recorded after superficial drying with absorbent paper. For each site, the AC index of feeding activity was calculated as the percentage of stomachs with food on the total number of examined stomachs. In order to evaluate the importance of the prey in the diet, three indexes were considered: percentage of occurrence (O%), the percentage of non-empty stomachs that contained a particular prey item; percentage by weight of prey (W%), calculated as the ratio of the weight of a prey category to the total weight of the stomach content; the index of relative importance (IRI%), modified by [28] as follows:
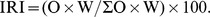



### Chemical and Biochemical Analyses

For each individual, liver and red muscle were separately extracted and organic extracts were analyzed in reverse phase high-performance liquid chromatography mass spectrometry (RP-HPLC-MS) to quantify algal metabolite.

Details of chemical analyses are reported in the supporting information file S1.

A battery of 12 biomarkers was chosen for evaluating the fish biochemical responses to the *C. racemosa* diet. The enzymatic activities of catalase, glutathione peroxidase and 7-ethoxyresorufin *O*-deethylase (EROD) were measured on all 45 samples, whereas the other biological responses (glutathione reductase, glutathione S-transferase, total glutathione, total oxyradical scavenging capacity, Acyl CoA oxidase, acetylcholinesterase, micronuclei frequencies, Na^+^-K^+^-ATPase), were evaluated on a subset of 18 individuals, 6 for each of three locations.

Details of these biochemical analyses are provided in the supporting information file S2.

### Statistical Analysis

Six separate PERMANOVAs [29], [30], one for each of the single response variables measured (CF, HSI, GSI, CAT, GPx and EROD activity) were employed to test the hypothesis of differences among fish assemblages from locations varying in terms of *C. racemosa* abundance. The design consisted of a single factor, namely Location (Lo), 3 levels, with n=15. In order to compare fish from the invaded locations with the non-invaded one, two levels of the Location factor (BR and TG) were contrasted *versus* the other (PC). The analysis was based on Euclidean distances, so the F-ratios used for tests in PERMANOVA were equivalent to those of traditional ANOVA. P-values were obtained using a permutation procedure, with 999 permutation units.

**Table 1:** Diet composition of *D. sargus.*

	AC=53.4		AC=73.4		AC=82.4	
	PC		TG		BR	
Prey items	IRI%	O%	IRI%	O%	IRI%	O%
*C. racemosa*	0.0	0.0	64.5	72.7	51.3	85.7
Articulated corallines	23.3	50.0	2.5	45.5	0.15	14.3
Bivalves	0.0	0.0	1.5	27.3	1.4	28.6
Chitonids	0.0	0.0	1.4	18.2	0.1	7.1
Gastropods	1.6	25.0	5.0	36.4	0.8	35.7
Sponges	3.2	12.5	12.3	27.3	1.9	21.4
Bryozoans	11.0	37.5	0.0	0.0	0.6	43.0
Polychaetes	49.7	37.5	0.1	9.1	7.2	21.4
Decapods	3.5	12.5	0.7	36.4	1.6	28.6
Ascidians	1.8	25.0	0.0	0.0	7.6	43.0
Amphipods	0.1	12.5	0.0	0.0	0.0	7.1
Unidentified	2.1	12.5	1.3	3.0	0.0	7.1

For each of the three study sites diet was expressed as percentage of frequency of occurrence (O%) and index of relative importance (IRI%). AC: % stomachs containing food.

PERMANOVA was also employed to test, in a multivariate context, the same hypothesis described above for the univariate analyses. In this case the analysis consider a matrix consisting of a battery of biomarkers (15 variables) and 18 samples (n=6) and was based on Euclidean distance measures calculated on the normalized data. Multivariate patterns of differences between fish were visualized by non-metric multidimensional scaling (nMDS) [31].

As PERMANOVA revealed significant differences for the contrast PC *vs* BR and TG and the nMDS showed that fish accumulating caulerpin were somewhat distinct from the others (see Results), a canonical analysis of principal coordinates (CAP) [32], [33] was performed to relate the caulerpin concentration in fish liver to the biotic resemblance matrix.

A further CAP ordination was then performed for the factor caulerpin set at three levels, namely absent, medium (between 7 and 26 µg g^−1^) and high (>47 µg g^−1^) to visualize differences among fish based on biological responses.

The contribution of each biomarker to differences seen in the second CAP plot was investigated by calculating product-moment correlations of original variables (biomarker responses) with canonical axes [33]. The correlations of individual variables with the two canonical axes (r_1_ and r_2_) were then represented as lines in the projection biplot. Biomarkers were included in the plot only if exceeding an arbitrarily chosen value of correlation (i.e. √(r_1_^2^+ r_2_^2^) ≥0.25).

Relation to caulerpin accumulation in fish liver in the nMDS and CAP ordinations, was visualized in 2d-bubble plots, by superimposing the values of metabolite concentration as circles of increasing size on the biotic ordination of the corresponding samples.

All analyses were done using the computer program PRIMER v6 [34], including the add-on package PERMANOVA+ [35].

## Results

### Diet Analysis and LC-MS Quantifications of Caulerpin in *D. sargus* Tissues

The feeding activity (AC) index ranged from 53.4% to 73.4% and 82.4% for fish collected in PC, TG and BR, respectively (Table 1).

**Table 2:** Concentrations of caulerpin in liver and red muscle of fish from BR and TG.

	Caulerpin (µg g^−1^)			Caulerpin (µg g^−1^)	
	liver	red		liver	red
BR1	0.0	0.0	TG1	7.1	0.0
BR2	12.6	25.4	TG2	0.0	0.0
BR3	42.8	16.0	TG3	0.0	0.0
BR4	20.9	38.2	TG4	56.2	18.1
BR5	0.0	0.0	TG5	25.9	20.5
BR6	0.0	0.0	TG6	0.0	0.0
BR7	20.5	37.7	TG7	115.1	124.6
BR8	10.1	14.8	TG8	7.4	8.8
BR9	8.1	0.0	TG9	187.7	222.8
BR10	0.0	0.0	TG10	10.6	0.0
BR11	25.9	110.2	TG11	0.0	0.0
BR12	13.4	68.5	TG12	0.0	0.0
BR13	55.0	28.8	TG13	0.0	0.0
BR14	32.4	38.8	TG14	8.4	0.0
BR15	46.6	94.9	TG15	9.5	5.1

Values of caulerpin concentrations are expressed per gram of dry weight (µg g^−1^).

**Table 3:** PERMANOVA testing differences in general condition markers of fish population among locations and between invaded (TG and BR) *vs*. non-invaded locations (PC).

		CF			HSI			GSI		
Source	df	MS	F	*p*	MS	F	*p*	MS	F	*p*
Lo	2	0.118	5.14	**	6.3E - 02	1.09	ns	2.6E - 02	0.4	ns
PC	1	7.5E - 02	2.88	ns	9.6E - 02	1.7	ns	3.09E - 03	4.7E - 02	ns
Res	44	2.3E - 02			5.81E - 02			6.57E - 02		

ns = not significant;

** *p*<0.01.

Eleven major food items were identified in the stomachs of *D. sargus* (Table 1). *Caulerpa racemosa* was the most important item in term of frequency of occurrence and relative importance in fish from TG and BR. The alga was absent in the fish from PC, where polychaetes, with the 49.7% of IRI, represented the main dietary component. For frequency of occurrence, bivalves, gastropods and decapods did not differ substantially in the diet of fish from TG and BR representing the following main items after *C. racemosa*. Following polychaetes, articulated corallines and bryozoans were the groups most represented in the diet of fish from PC (Table 1).

Chemical analysis revealed that 65% and 54% of fish from BR and TG accumulated caulerpin while no trace was detected in those from PC (Table 2). Caulerpin concentrations in liver ranged from 0 to 55.0 and from 0 to 187.7 µg g^−1^ dry weight for fish speared in BR and TG respectively; in red muscle, values ranged from 0 to 110.2 and from 0 to 222.8 µg g^−1^ dry weight for fish from BR and TG (Table 2).

### Statistical Analyses

**Figure 2:**
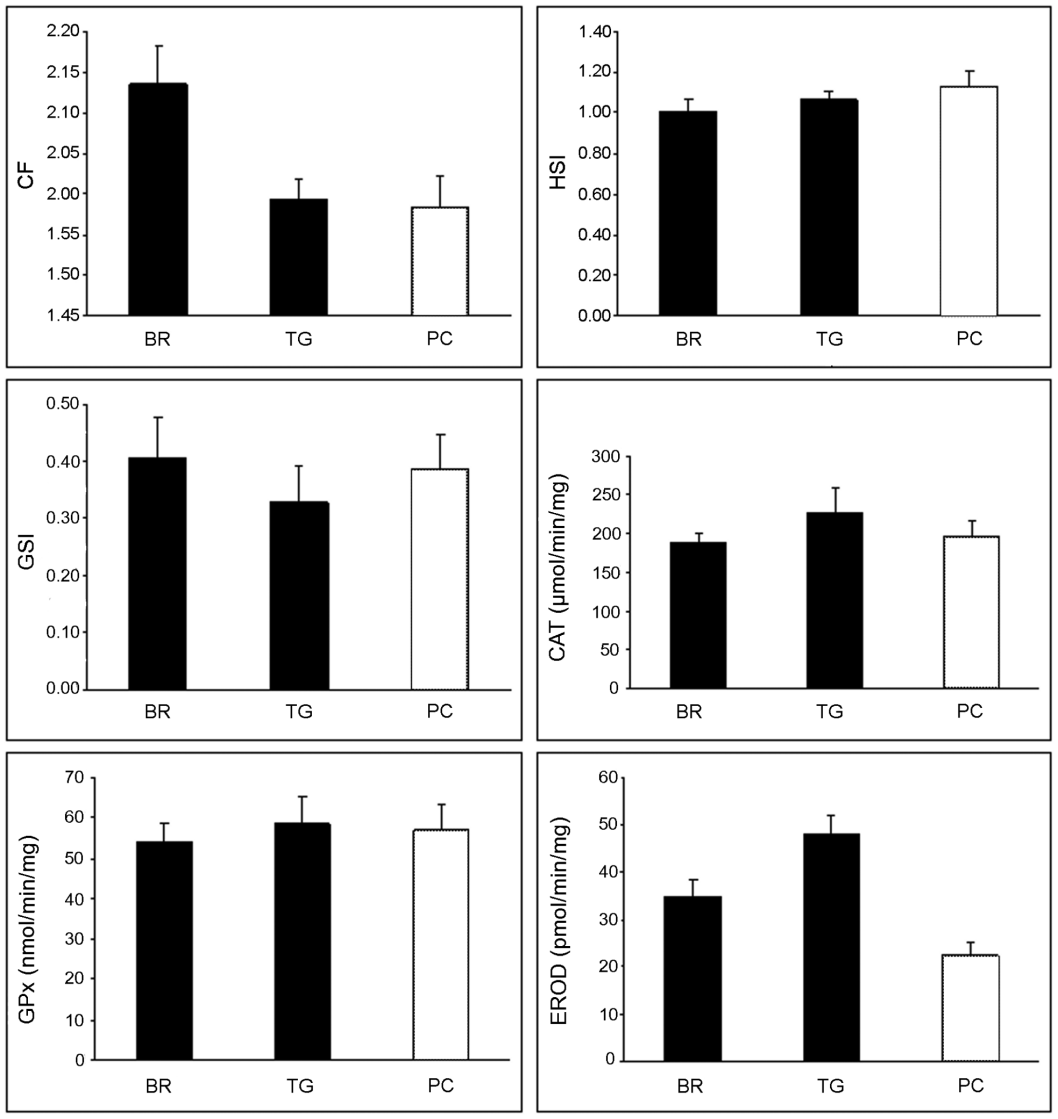
General condition markers and antioxidant biomarkers mean values for three study locations. Mean (±SE, n=15) of general condition markers (CF, HSI and GSI), antioxidant biomarkers (CAT and GPx) and activity of EROD were represented as a black bar for fish speared in the invaded locations (BR and TG) and white for non invaded area (PC).

PERMANOVA on the single response variables measured on 45 individuals, revealed no differences among locations for HSI, GSI and CAT (Table 3, 4). CF was significantly higher at BR with no differences between TG and PC (Table 3). Significant differences between invaded and non-invaded areas (i.e. PC *vs* (BR and TG) were detected for EROD (Table 4 and Figure 2).

**Table 4:** PERMANOVA testing differences in two antioxidant biomarkers (CAT and GPx) and activity of EROD of fish population among locations and between invaded (TG and BR) *vs*. non-invaded locations (PC).

		CAT			GPx			EROD		
Source	df	MS	F	*p*	MS	F	*p*	MS	F	*p*
Lo	2	6464	0.92	ns	80.27	0.15	ns	2415	12.32	***
PC	1	1247	0.18	ns	9.06	1.7E-02	ns	3509	15.9	***
Res	44	7002			548.6				195.98	

ns = not significant;

*** *p*<0.001.

**Table 5:** PERMANOVA investigating differences among fish populations across locations and between invaded (TG and BR) *vs*. non-invaded locations (PC).

Source	df	SS	MS	F	*p*
Lo	2	43.78	21.89	1.55	*
PC	1	33.03	33.03	2.38	**
Res	15	211.25	14.08		

Analysis based on Euclidean distance matrix of normalized multivariate dataset (18 individuals × 15 biomarker responses). Each test was performed using 999 permutations of appropriate units. ns = not significant;

** *p*<0.01.

**Figure 3:**
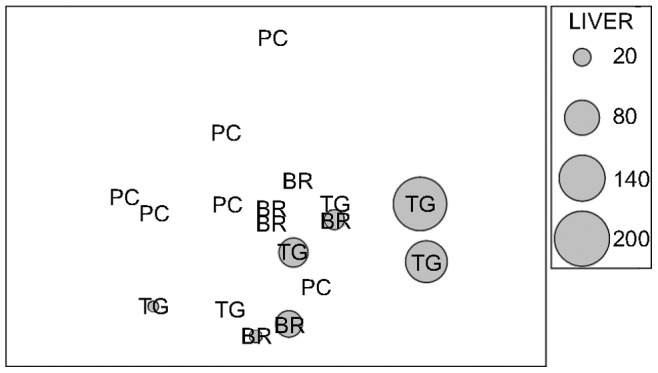
nMDS ordination of the Euclidean distance measure from normalized biomarker data. A subset of 18 individuals ×15 biomarker responses was used. On each individual, grey circles of increasing size with increasing caulerpin concentration in liver were superimposed.

**Figure 4:**
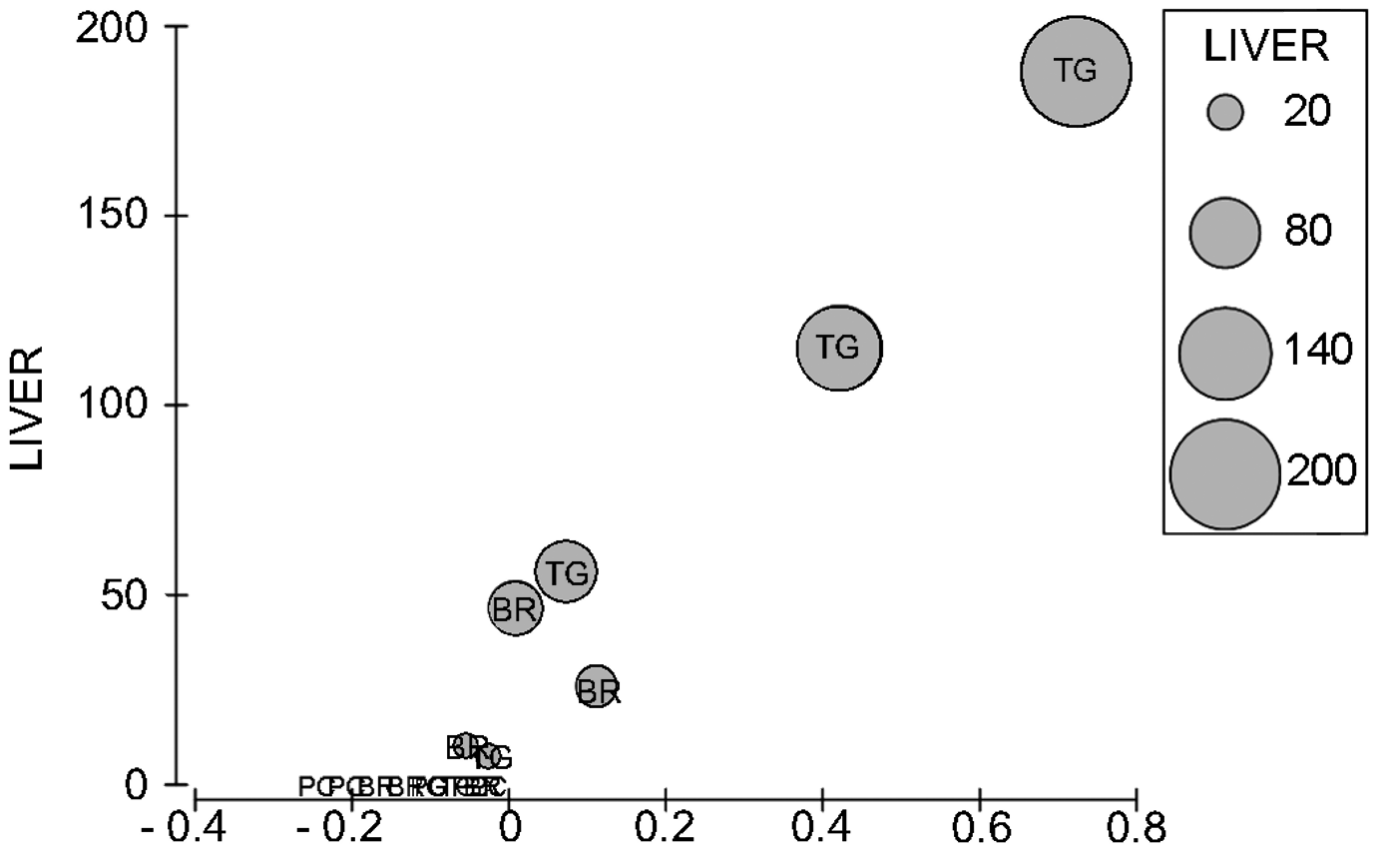
Canonical analysis of principal coordinates discriminating biochemical responses along caulerpin concentration gradient. CAP was based on the Euclidean distance matrix of the subset multivariate data. Caulerpin concentration in fish liver was represented with grey circles of increasing size superimposed on each specimen.

**Figure 5:**
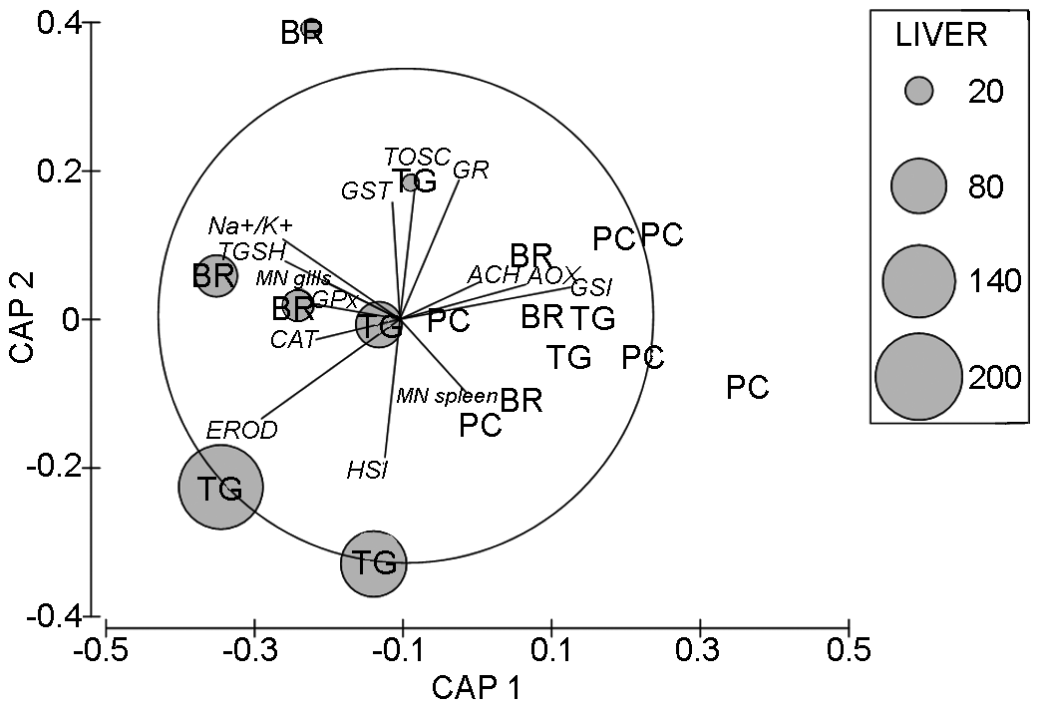
Canonical analysis of principal coordinates for the factor *Caulerpin.* CAP ordination was obtained from the distance matrix among fish assemblages on the basis of biological responses. Grey circles representing increasing caulerpin concentration in liver were superimposed on each individuals.

PERMANOVA on the multivariate dataset showed significant differences for the factor Locations and for the specific contrast PC *vs.* BR and TG (Table 5). Such a pattern was portrayed by the nMDS ordination (Figure 3), which segregated a group consisting of fish with the higher levels of caulerpin accumulation in the liver. This trend was confirmed by the canonical analysis of principal coordinates performed to relate fish biochemical responses to the alkaloid concentration (Figure 4) which explained, by using the first 11 axes, the 97.6% of variation of the biotic resemblance matrix, with a very high squared canonical correlation (δ_1_^2^=0.92). Fish separated according to a caulerpin accumulation gradient with individuals having accumulated this metabolite being placed to the right of the first axis of the plot.

The canonical analysis of principal coordinates for the term caulerpin achieved the highest allocation success (66.7%) using m=8 principal coordinate (PCO) axes, which explained the 88.9% of variation of the resemblance matrix.

The first canonical axis had very high canonical correlations with the multivariate data (δ_1_^2^=0.74). Fish accumulating caulerpin were clearly distinct along the first axis from those without metabolite in their tissues that, in turn, clustered together on the right-hand side of the graph (Figure 5). The second axis (δ_2_^2^=0.43) separated fish with higher caulerpin concentration from those with low amounts.

Several biomarkers were highly correlated with the canonical axes. More specifically, EROD, catalase, glutathione peroxidases, total glutathione and Na^+^-K^+^-ATPase contributed to separate fish having accumulated caulerpin from the others, while gluthathione S-transferases and TOSC values were more effective in discriminating between fish with higher and lower levels of caulerpin. Acyl CoA oxidase (AOX) and acetylcholinesterase activities were, on the contrary, strongly related to fish without caulerpin.

The frequency of micronuclei in gills was higher in fish with caulerpin, but an opposite, weaker correlation, was observed in spleen tissues (Figure 5).

Among general condition markers, specimens with high levels of caulerpin in the liver showed a strong correlation with HSI, whereas higher values of GSI were measured in individuals not accumulating the algal metabolite.

## Discussion

Invasive species can interact negatively with native ones by altering availability or quality of nutrients, food and physical resources, changing habitat structure and affecting gene flow or reproductive performance [3]–[5], [36]. Whereas the effects of *C. racemosa* invasion on seabed and benthic community has been widely described [37]–[43], this study represents a first multidisciplinary approach towards the understanding of the effects of *C. racemosa* invasion on habitat use and diet composition of the native fish species *D. sargus. Caulerpa racemosa* has become a major food item in the diet of this important fish species [22]. Here, we confirm the frequent occurrence of invasive alga in stomach contents of the fish with the concomitant accumulation in fish tissues of the caulerpin. The switch from a diet composed of animal and plant items to a diet based mostly on the invasive alga, could influence organoleptic properties and nutrition quality of this economically important fish resource. The nutritional value, taste and flavour of the fish fillet in fact, depend both on the amount of fat and fatty acid composition and on the muscle amino acids which are all strongly influenced by the dietary history [44].

The occurrence of biochemical perturbations in fish consuming the pest alga was showed. By using biomarkers, we, indeed, found that exposure to the alga induced stress conditions, increased metabolic activity of detoxification and, also, changes in the morphology of gross gonads.

Specifically, the high exposure to the alga, deduced by the high accumulation of caulerpin, seems to trigger the activity of 7-etoxyresorufin *O*-deethylase (EROD), suggesting the involvement of cytochrome P450 biotrasformation pathway in the metabolism of *Caulerpa*.

Cyt P450 plays a key role in metabolism, providing resistance to many organic xenobiotics. However, Cyt P450 can increase intracellular oxyradical formation and activate certain chemicals to mutagenic metabolites, thus enhancing the likelihood of carcinogenicity [45], [46].

Relationship between elevated Cyt P450 activity and decreased reproductive success (e.g. egg survival, fertilization success and hatchability) are known [47], thus suggesting potential negative effects of algal compounds on fish reproductive success acting through the induction of this pathway or related oxidative stress conditions.

Oxidative stress is an important general toxicity pathway for many xenobiotics and biologically active compounds [48]–[51] and also *Caulerpa* spp. have been recently demonstrated to increase production of ROS in fish, leading to oxidative damage [10], [17], [22]. Prooxidant effects of *C. racemosa* based-diet on white sea breams have been already suggested by [22] by the significant modulation in the activity of catalase and glutathione peroxidases. The activation of antioxidant defenses is further supported here both in terms of induction of more sensitive oxidative biomarkers, i.e., glutathione S-transferases (GST) and glutathione, and as overall capability to neutralize ROS indicated by the Total Oxyradical Scavenging Capacity (TOSC). On the other hand, the greater activity of GST in fish with lower caulerpin accumulation might suggest a biphasic response of these enzymes, as previously shown for several antioxidants defences which, after an initial counteracting response, can be inhibited above a certain oxidative pressure [52]. Beside the reduction of organic hydroperoxides, glutathione-S-transferases are involved in biotransformation of electrophilic organic compounds metabolized by cytochrome P-450 [53]; several conflicting responses have been reported in field conditions according to intensity and duration of exposure [54]. Glutathione reductase, involved in the conversion of oxidized glutathione (GSSG) to its reduced form (GSH), did not appear to be influenced by caulerpin accumulation, suggesting that this response is not primarily involved in oxidative responses to *C. racemosa* ingestion.

During biotransformation and oxidative processes, highly reactive intermediates can be produced and interact with DNA, leading to a series of measurable alterations (e.g. point mutations, chromosomal re-arrangements, DNA adducts, DNA strand breaks and increased number of micronuclei) [55]. Considering the importance of effects associated with DNA damage, genotoxicity biomarkers are considered particularly important for identification of potential risk and adverse health effects. Here we used micronucleus-frequencies as genotoxicity biomarker, which enabled us to evaluate direct effects of algal compounds on the loss of DNA integrity.

MN frequency in gills was positively related to the presence of algal metabolites in fish tissues, whereas the same biomarker measured in spleen was weakly related with groups of fish not accumulating caulerpin. The different response of MN between spleen and gills observed in this study is consistent with the greater sensitivity to genotoxic injury of gill epithelium cells than other tissues as proved in several studies [e.g. 56,57].

Genotoxic damage may result in several physio-pathological modifications described by [58] and referred to as ";genotoxic disease syndrome";. These modifications could result in detrimental effect on metabolism and on cell integrity with finally fitness reduction. Marked effects on population dynamics are expected when such reduction result in a decrease of size of individuals at levels beneath the sustainability [55], [59]. If direct genotoxic effects are exerted on germinal cells, the resulting alterations could be transmitted to the future generations. Heritable mutations could result either in impairment in matching or in non-viable offspring, also in this case eventually reducing the overall population fitness. Therefore, further analyses are needed to evaluate what type of cells, somatic or germinal, are affected by *C. racemosa* metabolites in order to foresee evolutionary consequences on *D. sargus* populations.

Na+-K+-ATPase pump is responsible for the electrochemical gradient across the plasma membrane and critical for the osmotic balance of the cell, the resting membrane potential, and the excitable properties of muscle and nerve cells. Previous in vitro studies demonstrated the sensitivity of Na+-K+-ATPase activity of leech neurons to caulerpa metabolites, with a particular inhibition by the terpenoid caulerpenyne [15], [16]. In contrast to results obtained in in vitro experiments, in the present work Na+-K+-ATPase appeared to strongly increase in fish from the locations invaded by C. *racemosa*. These results suggest a different species-specific sensitivity of this enzyme to caulerpa metabolites, but also highlight the caution needed in extrapolating *in vivo* effects from *in vitro* results.

Acetylcholinesterase (AChE) is pivotal for the proper functioning of nervous system of vertebrates and invertebrates. Inhibition of this enzyme increases the concentration of acetylcholine (ACh) in the synaptic cleft, causing continued stimulation of neurons and suppression of neurotransmission to organs [60]. It has been suggested that this biomarker may represent an useful early warning of potential effects on populations, since positive relationships between reduced levels of AChE and increased mortality and reduced abundance were observed in microcosms [61].

Despite organophosphate and carbammate pesticides are well known inhibitors of AChE, other compounds have been recently shown to influence this enzymatic activity, such as metals [62] detergents [63] and algal toxins [64]. [65] showed that the indole moiety in alkaloids derived from the stem of a plant was liable for inhibition of AChE activities. Specifically, docking trials showed the existence of an interaction between 1H indole and the active site of AChE, leading to a good enzymatic inhibitory activity.

Caulerpin has a molecular structure very similar to the active ones studied by [65] with two 1H indole moieties linked together by an eight-membered cyclooctatetraene ring [66]. Lower levels of AChE observed in fish accumulating caulerpin might support an involvement in neurotoxic effects. The descriptive nature of this study does not allow us to infer too much on the inhibitory mechanism of the algal metabolites, but the presence of two indole moieties in the molecular structure of caulerpin would be in agreement with the results reported by [65].

Analyses of AOX revealed a reduced peroxisomal proliferation in fish with a higher content of caulerpin. However, such variations may not necessarily reflect an inhibitory effect of the algal metabolite, but simply the different diet and fatty acid composition. Previous studies confirmed a marked variability for AOX activity related to feeding behaviour and to different availability of substrates such as prostaglandins and leukotriens [67].

The individual gross indices are relatively easy to measure and can reflect adverse effects at the organism level [68]. A probable status of liver hypertrophy in relation to the exposure to *C. racemosa* has been already suggested by [22]. HSI is sensitive to the nutritional status of the animal and gives an useful indication of energy reserves. Liver enlargement in breams can be caused by enhanced biotransformation requirement of exogenous molecules and positive correlations between HSI and concentrations of lipophilic contaminants have been widely reported [69]–[72]. The same relationship observed in the present investigation could confirm the possibility of liver hypertrophy in animals ingesting *C. racemosa* and inducing the cytochrome P450 pathway.

All the sampled fish showed sexual maturation as assessed by visual analysis of the gonads according to [73], [74]. In contrast with what was reported in [22] where no significant relationship was found between GSI and levels of caulerpin in fish tissues, this study, based on an higher sample size and a structured sampling design, highlighted GSI as significantly lower in fish exposed to invasive alga. GSI is frequently reported as a general measure of gonad maturation and spawning readiness, based on the broad assumption that proportionally larger gonads indicate greater development [75]. The significant relation between GSI and fish not accumulating caulerpin lead us to hypothesize a potential detrimental effect of the *Caulerpa*-based diet on gross gonadal morphology.

*D. sargus* reproduces once a year [74], and shows a similar gonad development for males and females. The spawning period changes with the latitude, starting earlier at lower latitudes [74], [76]. [77] showed that in Italy the majority of fish are post-spawner in June, resting during July and in recrudescing phase from August to September. In this respect, a potential activity of algal metabolites on the fish gonads is more likely to occur during the development stage of gonads (as in the present study study) rather than during the resting phase.

By using biomarkers we have evaluated early warning signals of biological responses at the subcellular level, from biotransformation and oxidative challenge, to genotoxic endpoints, plasma membrane potential, cholinergic transmission and peroxisomal proliferation. Such deviation from normal ranges of biochemical responses could have, in long-term perspective, great biological and ecological consequences. Detoxification of xenobiotics is a process that requires high levels of metabolic resources. Energy spent in detoxification can not be used for storage, growth and reproduction. Prolonged exposure to algal compounds could have then complex effects on the supply and demand of metabolism and hence on fitness, growth, well-being and survival.

The alteration of cholinergic transmission, here measured as acetylcholinesterase activity, might impair survival by affecting daily activity performance such as feeding, predator avoidance and swimming.

The exposure to ROS could cause degenerative processes or atrophy of tissues and organs, impairments of immunoresponse and reproduction, premature aging and lower survival rate.

Finally, although few data on temporal changes in fish density are actually available for the studied locations, future effects on reproductive capacity could be hypothesised as long term effects, leading to possible decline of *D. sargus* population.

### Conclusions

In spite of the observed cellular and physiological alterations, *C. racemo*sa has become the preferred food for *D. sargus* in the invaded areas, suggesting a possible involvement of the algal metabolites in the physiological control of food intake. This gives urgency to further studies performed on captive animals under controlled laboratory conditions aimed at exploring the influence of selected chemical signals on the appetite-regulatory systems of the Mediterranean white seabream.

Further investigations are also needed to evaluate, by manipulative experiments, the link between particular algal metabolites with reproductive performance of fish, their changes in biochemical responses and their potential effects at a population level. The entry of algal toxic metabolites in trophic webs could permanently alter biochemical cellular processes, acting also during vulnerable periods of organism development, eventually leading to changes at the population and possible community levels of biological organization.

These effects could also interact with other multiple stressors (such as anthropogenic pollutants, viral diseases), potentially affecting habitat protection programs and the real target of conservation initiative. Results of this study could provide new perspectives to the analyses of fishery resources usually modelled only in relation to overexploitation and provide to managers of MPAs and regulators new elements to be considered for a sustainable management of coastal habitats and species.

## Supporting Information

File S1 Details of chemical analyses for extraction and quantification of algal metabolite.
(DOC)

File S2 Details of biochemical analyses.
(DOC)
