# Supplementary material for: Subtle Effects of Biological Invasions: Cellular and Physiological Responses of Fish Eating the Exotic Pest Caulerpa racemosa
Source: PLoS One. 2012 Jun 11;7(6):e38763. doi: 10.1371/journal.pone.0038763 (PMC3372483; doi:10.1371/journal.pone.0038763)
Supplement: File S1 — Details of chemical analyses for extraction and quantification of algal metabolite. (DOC) [file pone.0038763.s001.doc]

**Supporting Information File S1**

**Extraction and quantification of algal metabolite in fish tissues:** **details of chemical analyses**

Liver and red muscle were extracted with acetone by both ultrasound and homogenizing with a pestle. The extracts were filtered on paper, and the clear filtrates were evaporated under vacuum. After removing the organic solvent, the aqueous residues were further extracted 3 times with ethyl acetate. Organic extracts were dried under nitrogen and reconstituted in MeOH at a final concentration of 0.5 mg ml^–1^. Reverse phase high-performance liquid chromatography mass spectrometry (RP-HPLC-MS) analyses were carried out on a C-18 Gemini column (Phenomenex, 150 × 2.0 mm, 5 μm) at room temperature.

The injection volume was 20 μl. The mobile phase consisted of a MeOH:H_2_O gradient from 70:30 to 90:10 in 15 min (flow 0.2 ml), monitoring peak elution with a photodiode array (PDA) detector, coupled with a Q-Tof *micro* mass spectrometer (Waters) in electrospray ionization (ESI)-positive ion mode. The caulerpin calibration curve was prepared in methanol by adding known amounts of caulerpin (for details of acquisition of caulerpin standard see [1]). It consisted of 2 blank samples and 5 calibration points (in triplicate) at concentrations ranging from 10 to 10 000 ng ml^–1^. The resulting peak areas were plotted against the concentrations.

**Reference**

1. Terlizzi A, Felline S, Lionetto MG, Caricato R, Perfetti V et al. (2011) Detrimental physiological effects of the invasive alga *Caulerpa racemosa* on the Mediterranean white seabream *Diplodus sargus*. Aquat Biol 12: 109–117
