# Supplementary material for: Subtle Effects of Biological Invasions: Cellular and Physiological Responses of Fish Eating the Exotic Pest Caulerpa racemosa
Source: PLoS One. 2012 Jun 11;7(6):e38763. doi: 10.1371/journal.pone.0038763 (PMC3372483; doi:10.1371/journal.pone.0038763)
Supplement: File S2 — Details of biochemical analyses. (DOC) [file pone.0038763.s002.doc]

**Supporting Information File S2**

**Details of biochemical analyses**

EROD was measured according to [1] in the S9 fraction of individual livers homogenized (1:5, w/v) in 100 mM K-phosphate buffer pH 7.5, 0.15 M KCl, 1 mM ethylenediaminetetraacetic acid (EDTA). After centrifugation at 12000 g for 15 min, the resulting supernatants (S9) were immediately incubated at 30°C in a final volume of 1 ml containing 100 mM K-phosphate buffer pH 7.5, 4 mM 7-ethoxyresorufin, and 0.25 mM β-nicotinamide adenine dinucleotide (NADPH); 2 ml acetone were added after 5 min to stop the reaction. Incubation mixtures stopped at time zero were used as blank values and were subtracted from the sample fluorescence. Fluorimetric analyses (535/585 nm) were quantified by reference to resorufin standards (0.02–1 μM).

Variations of the main antioxidants were measured in liver cytosolic fractions after homogenization (1:5, w/v) in 100 mM K-phosphate buffer pH 7.5, 0.1 mM phenylmetylsulfonilfluoride (PMSF), 0.5 μg ml^-1^ pepsatin, NaCl 1.8%, and centrifugation at 100000 g for 1 h at 4 °C. Catalase (CAT) was analysed by the consumption of H_2_O_2_ at 240 nm (ε = -0.04 mM/cm); the assay concentration was 12 mM H_2_O_2_ in 100 mM K-phosphate buffer pH 7.0. Glutathione reductase (GR) was quantified by the loss of NADPH during the reduction of oxidized glutathione GSSG (λ =340 nm, ε = -6.22 mM cm^-1^); the final assay conditions were 100 mM K-phosphate buffer pH 7.0, 1 mM GSSG, and 0.12 mM NADPH. Glutathione S-transferases (GST) were measured at 340 nm, in 100 mM K-phosphate buffer pH 6.5, with 1.5 mM reduced glutathione, and 1.5 mM 1-chloro-2,4-dinitrobenzene (CDNB) as substrate (ε = 9.6 mM cm^-1^). Total glutathione (TGSH) was analysed in samples homogenized (1:5, w/v) in 5% sulfosalicilic acid with 4 mM EDTA, maintained for 45 min on ice and centrifuged at 37000 g for 15 min. The resulting supernatants were enzymatically assayed by the enzymatic method of [2].

Glutathione peroxidases (GSH-Px) were assayed by the method of [3] on samples, obtained by homogenizing the tissue in 1:5 (tissue w/v) ratio in 10 mM ice-cold sodium phosphate buffer containing 1 mM dithiothreitol (DTT) (pH 7). The assay mixture consisted of phosphate buffer (NaH_2_PO_4_ 50 mM, EDTA 0.4 mM, pH 7), 1 mM sodium azide, 1 IU ml^-1^ glutathione reductase, 1 mM GSH, 0.2 mM NADPH, 0.25 mM H_2_O_2_ in a total volume of 2 ml. Oxidation of NADPH was recorded spectrophotometrically at 340 nm. The enzyme activity was calculated as nmol NADPH oxidized min^-1^ mg^-1^ of proteins.

The total oxyradical scavenging capacity (TOSC) was determined in liver cytosolic fractions obtained as previously reported without PMSF in the homogenization buffer [4]. The TOSC assay measures the overall capability of cellular antioxidants to absorb different forms of artificially generated oxyradicals, thus inhibiting the oxidation of 0.2 mM α-keto-γ-methiolbutyric acid (KMBA) to ethylene gas [5,6]. Peroxyl radicals (ROO˙) were generated by the thermal homolysis of 20 mM 2-2'-azo-bis-(2methylpropionamidine)-dihydrochloride (ABAP) in 100 mM K-phosphate buffer, pH 7.4. Hydroxyl radicals (˙OH) were produced by the Fenton reaction of iron-EDTA (1.8 μM Fe3+, 3.6 μM EDTA) plus ascorbate (180 μM) in 100 mM K-phosphate buffer. Ethylene formation in control and sample reactions was analysed by gas-chromatographic analyses according to [6]. The TOSC values were quantified from the equation: TOSC = 100 - (∫SA/∫CA × 100), where ∫SA and ∫CA are the integrated areas calculated under the kinetic curves for samples (SA) and control (CA) reactions. For all the samples, a specific TOSC (normalized to content of protein) was calculated by dividing the experimental TOSC values by the relative protein concentration contained in the assay.

The peroxisomal enzyme Acyl CoA oxidase was analysed in liver samples homogenized in 1 mM sodium bicarbonate buffer (pH 7.6), containing 1 mM EDTA, 0.1% ethanol, 0.01% Triton X-100 and centrifuged at 500 g for 15 min at 4 °C. The H_2_O_2_ production was measured in a coupled assay [7,8] by following the oxidation of dichlorofluorescein-diacetate (DCF-DA) catalyzed by an exogenous horseradish peroxidase (HRP). The reaction medium was 0.5 M K-phosphate buffer (pH 7.4), 2.2 mM DCF-DA, 40 μM sodium azide, 0.01% Triton X-100, 1.2 U ml^-1^ HRP in a final volume of 1 ml. After a pre-incubation at 25 °C for 5 min in the dark with an appropriate volume of sample, reactions were started adding the substrate palmitoyl-CoA at a final concentration of 30 μM. Readings were carried out against a blank without substrate at 502 nm.

Acetylcholinesterase (AChE) activity was analysed in white muscle homogenized in 0.1 M Tris–HCl buffer pH 7.2, 0.25 M saccarose and centrifuged at 10 000 g for 10 min, according to the method described by [9]. Obtained supernatants were spectrophotometrically assayed by the Ellman’s reaction at 18 ± 1 °C, λ = 412 nm, ε = 13.6 mM cm^-1^.

Na^+^-K^+^-ATPase activity was analysed in white muscle homogenized in MgCl_2_ 5 mM, NaCl 150 mM, NH_4_Cl 100 mM, Tris 20 mM, pH 7.3 with HCl. The inorganic phosphate appearance in the reaction medium in the presence and absence of ouabain was measured. Briefly, homogenate samples (75 µg of proteins) were added to 500 µl of buffer A containing in MgCl_2_ 5mM, NaCl 150 mM, KCl 100 mM, Tris 20 mM, pH 7.3 with HCl or buffer B (buffer A plus 4.8 mM ouabain). The enzymatic reaction was followed for 10 min at 25°C and then stopped by boiling. The samples were then centrifuged (15290 g × 10 min) in order to precipitate denaturated protein complexes. Inorganic phosphate was determined on the resulting supernatant by using the method described by [10]. The enzymatic activity was expressed as micromoles of inorganic phosphate released in 1 min per milligram of proteins.

Micronuclei (MN) frequencies were measured in cell suspensions from spleen and gills rapidly washed in saline buffer, fixed in Carnoy’s solution (3:1 ethanol, acetic acid), dispersed on glass slides and stained with the fluorescent dye 40,6-diamidino-2-phenylindole (DAPI) at 100 ng ml^-1^. For each specimen, 2000 cells with preserved cytoplasm were scored for the presence of micronuclei, defined as round structures, smaller than 1/3 of the main nucleus diameter, on the same optical plan and clearly separated from it [11].

**References**

1. ICES (1998) In: Stagg R, McIntosh A, editors. Biological effects of contaminants: determination of CYP1A-dependent Mono-oxygenase activity in Dab by fluorometric measurement of EROD activity, vol 23. ICES Techniques in Marine Environmental Sciences. p. 16
2. Akerboom TPM, Sies H (1981) Assay of glutathione, glutathione disulfide and mixed disulfides in biological samples. Meth Enzymol 71: 373–382
3. Wendel A (1980) Glutathione peroxidase. In: Jakoby WB, editor. Enzymatic basis of detoxification. New York: Academic Press. pp. 333–353
4. Regoli F, Winston GW, Gorbi S, Frenzilli G, Nigro M et al. (2003) Integrating enzymatic responses to organic chemical exposure with total oxyradical absorbing capacity and DNA damage in the european eel *Anguilla anguilla*. Environ Toxicol Chem 22: 2120–2129
5. Winston GW, Regoli F, Dugas AJ, Fong JH, Blanchard KA (1998) A rapid gas chromatographic assay for determining oxyradical scavenging capacity of antioxidants and biological fluids. Free Radical Bio Med 24: 480–493
6. Regoli F, Winston GW (1999) Quantification of total oxidant scavenging capacity of antioxidants for peroxynitrite, peroxyl radicals, and hydroxyl radicals. Toxicol Appl Pharm 156: 96–105
7. Orbea A, Ortiz-Zarragoitia M, Solé M, Porte C, Cajaraville MP (2002) Antioxidant enzymes and peroxisome proliferation in relation to contaminant body burdens of PAHs and PCBs in bivalve molluscs, crabs and fish from the Urdaibai and Plentzia estuaries (Bay of Biscay). Aquat Toxicol 58: 75–98
8. Bocchetti R, Fattorini D, Pisanelli B, Macchia S, Oliviero L et al. (2008) Contaminant accumulation and biomarker responses in caged mussels, *Mytilus galloprovincialis*, to evaluate bioavailability and toxicological effects of remobilized chemicals during dredging and disposal operations in harbour areas. Aquat Toxicol 89: 257–266
9. Ellman GL, Courtney KO, Anders V, Featherstone RM (1961) A new and rapid colorimetric determination of acetylcholinesterase activity. Biochem Pharmacol 7: 88–95
10. Higgins AJ (1978) Separation and analysis of membrane lipids components. In: Findlay JBC, Evans WH, editors. Biological membranes, a practical approach. Oxford: IRL Press. pp. 103–107
11. Nigro M, Falleni A, Barga ID, Scarcelli V, Lucchesi P et al. (2006) Cellular biomarkers for monitoring estuarine environments: transplanted versus native mussels. Aquat Toxicol 77: 339–347
